# Prevalence and factors associated with insomnia among medical students in China during the COVID-19 pandemic: characterization and associated factors

**DOI:** 10.1186/s12888-023-04556-8

**Published:** 2023-03-07

**Authors:** Ming Zhang, Li Qin, Dongmei Zhang, Mingfen Tao, Kangrong Han, Chenru Chi, Zhongying Zhang, Xiubin Tao, Huan Liu

**Affiliations:** 1 School of Innovation and Entrepreneurship, Wannan Medical College, Wuhu, 241002 Anhui China; 2 Academic Affairs Office, Wannan Medical College, Wuhu, 241002 Anhui China; 3 Department of Nursing, Wannan Medical College, Wuhu, 241002 Anhui China; 4 Department of Hemodialysis, The First Affiliated Hospital of Wannan Medical College (Yijishan Hospital of Wannan Medical College), Wuhu, 241001 Anhui China; 5 Ye Peida College of Innovation and Entrepreneurship, Beijing University of Posts and Telecommunications, Beijing, 100876 China; 6 Graduate School of Wannan Medical College, Wuhu, 241002 Anhui China; 7 School of Management Engineering, Anhui Institute of Information Technology. Wuhu, Anhui, 241199 China; 8 Department of Nursing, The First Affiliated Hospital of Wannan Medical College (Yijishan Hospital of Wannan Medical College), Wuhu, 241001 Anhui China

**Keywords:** Insomnia, COVID-19, Epidemic, Anxiety, Depression, Medical students

## Abstract

**Background:**

Insomnia has become an important issue in recent years. Insomnia is affected by many factors. Previous research has shown that during the COVID-19 pandemic, there would be a long-term negative effect on the mental health of medical college students. The state of medical college students’ insomnia directly determines the results of medical education and the career development prospects of the medical students themselves. Therefore, it is very important to understand the insomnia situation of medical students in the post-epidemic era.

**Methods:**

This study was conducted 2 years after the global COVID-19 pandemic (April 1-April 23, 2022). The study used an online questionnaire, administered through a web-based survey platform. The Athens Insomnia Scale (AIS), Fear of COVID‐19 Scale (FCV-19S), GAD-2, PHQ-2, and socio-demographic information were surveyed by the Questionnaire Star platform.

**Results:**

The prevalence of insomnia was 27.80% (636/2289). Grade(*P* < 0.05), age(*P* < 0.001),

loneliness(*P* < 0.001), depression(*P* < 0.001), anxiety(*P* < 0.001), fear of COVID-19 was highly correlated with insomnia (*P* < 0.001). Adapting to online class(*P* < 0.001) was a protective factor of smartphone addiction.

**Conclusions:**

This survey shows that Insomnia was highly prevalent among the Chinese medical college students during the COVID-19 pandemic. Governments and schools should through psychological interventions to address the current situation of insomnia among medical students, and formulate targeted programs and strategies to reduce their psychological problems.

## Introduction

Sleep is one of the important pillars of human health and the function of maintaining human life [1]. Healthy and adequate sleep is necessary to maintain individual physical and mental health as well as good emotional and social functioning, while its insomnia may have many adverse consequences [2]. Insomnia is defined as dissatisfaction with the quality or quantity of sleep [3]. Insomnia is one of the most common sleep disorders which can adversely affect an individual's quality of life and mental health, and is associated with various physical complications, such as increased risk of obesity, diabetes, heart disease and stroke [4]. The prevalence of insomnia was reported to be 18.5% among Chinese college students [5]. Insomnia is great clinically significant as it significantly impairs the physical and mental health of individuals [6]. Insomnia has been shown to be a prominent public health problem with medical college students in particular. Previous research has shown that insomnia may be significantly related to memory reduction and decreased learning abilities, ultimately leading to a decline in their academic performance among medical students [7, 8]. More worryingly, decreased sleep quality increases the likelihood that individuals will develop psychiatric disorders (such as anxiety and depression), and may develop suicidal behavior [9].Medical students have mastered more professional medical knowledge and are an important backup force in the medical and health industry [7]. As the backup force of future medical staff, the insomnia problem of medical students needs to be paid more attention. Previous studies have shown that prevalence of insomnia is higher among undergraduate medical students compared to undergraduates in other majors [10]. At the same time, medical college students are one of the special groups that have received great attention in the post-epidemic era, the COVID-19 pandemic could have a huge mental health impact on them, which could be attributed to the growing concerns about academic performance and reduced social and recreational activities [11].

The COVID-19 pandemic has presented medical students with many serious academically challenges, and university life has become more stressful than usual for many medical students. Medical college students are known to experience enormous stress during the new COVID-19 pandemic, due to overwork, the high risk of infection, lack of contact with family, and isolation during COVID-19 [12]. As a result of the COVID-19 pandemic, medical students feel more confused about their future careers, which can lead to poor mental health problems such as fear and insomnia [13]. The main COVID-19 control practices include wearing masks, hand washing, avoiding crowded places, and social distance [14]. Studies have shown that social distancing was necessary to effectively control the COVID-19 pandemic, it also has a severe negative impact on college students' sleep quality and sleep behavior alongside a consensus on the literature [11]. Due to the COVID-19 pandemic, college students must be confined to residence halls, with social distancing and self-isolation limiting their interactions with teachers and classmates. Face-to-face offline classroom instruction has shifted to online class to avoid direct close contact between teachers and students, and difficulty adjusting to online courses makes college students feel more fearful, anxious, and depressed [15]. Students’ mental health problems of mild to severe symptoms of depression, anxiety and stress ranged from 46.92% to 82.4%, 26.6% to 96.82%, and 28.5% to 70.1%, respectively, whereas meta-analytic study reported that students were experiencing a higher rate of depression, anxiety, and stress than general people and healthcare professionals [16]. With the dramatic increase in the number of asymptomatic cases of COVID-19 and heightened uncertainty about the outbreak, medical students will also be under increasing pressure due to online learning.

College students, as a special group, may have experienced a greater psychological impact from the COVID-19 pandemic, which could be due to increased academic performance concerns in online learning and drastic reduction social activities [11]. According to previous studies, college students are more vulnerable to various impacts from sudden changes in the COVID-19 pandemic [17]. These constant COVID-19 pandemic-related stressors may adversely affect the sleep quality of medical students. During the COVID-19 pandemic, the mental health of college students has been affected to varying degrees [18]. Satici B et al. found that fear of COVID-19 may lead to severe psychological problems in individuals, which in turn may reduce people's satisfaction with their lives during the COVID-19 pandemic [19]. During the new wave of the COVID-19 pandemic, the pressure of strict quarantine measures and the academic pressure of school closures made medical students more prone to insomnia problems [20]. Based on the above-mentioned research evidence of the impact of COVID-19 on mental health, it is reasonable to speculate that insomnia status of medical students may also be severely affected during the new outbreak of COVID-19. As it has been more than 2 years since the COVID-19 pandemic, insomnia in college students is an important research area, and thus this issue increasingly gaining global attention.

To our knowledge, at the time of writing this research paper, no studies have examined insomnia among medical students during the new wave of COVID-19 pandemic in China. By understanding the impact of the COVID-19 outbreak on medical students, insomnia and studying the influencing factors underneath, effective interventions can be developed and provided for medical students to improve their insomnia. Therefore, using a web-based, single-center, large-sample cross-sectional study, we aimed to assess the insomnia status of Chinese medical students during the COVID-19 outbreak, and to explore the possible influence factors. We hope that our study results will provide data support for the targeted interventions on insomnia among Chinese medical students after the COVID-19 pandemic. The study was conducted a pilot study with a small number of participants before the survey began to determine participants, understanding of the survey questions.

## Materials and methods

### Study design

Our research group designed a cross-sectional study to investigate the insomnia status of the Chinese medical students during the new wave of COVID-19 pandemic using an anonymous and confidential online questionnaire. Stratified cluster sampling was used, with grade as the main sampling unit. We selected four grades, namely, first, second, third and fourth grade.

### Study participants

This is a cross-sectional study conducted through an online survey conducted from April 1 to April 23, 2022. The investigation period corresponded to the new wave of the COVID-19 pandemic in China. We distributed self-administered questionnaires to university students at Wannan Medical College through an app called Wenjuanxing (www.wjx.cn). The research group developed an electronic consent form and a questionnaire using the shared Questionnaire Star platform. The inclusion criteria were as follows: being a student of Wannan Medical College and willingness to participate in this research. The exclusion criteria were students not studying at Wannan Medical College. The survey contents included Socio-Demographic Data, insomnia, anxiety, depression, COVID-19 fear, loneliness and e-health literacy. Various professional counselors and student leaders assisted in this survey and help to recruit the participants. Before data collection, the research's process and objectives were explained to the medical college students in detail, and signed the electronic informed consent form after confirming their voluntary to participation. The participants were informed before the questionnaires that all information provided with the study would be kept confidential and the data would be anonymous. Also the participants could free to withdraw from the study at any time during the data collection process. Participants can only be able to effectively submit the questionnaire if they have answered all the content of the questionnaire. Finally, a total of 2289 questionnaires were collected and included in the data analysis.

### Data collection procedure

The online questionnaire survey was conducted on the basis of an electronic online platform called "Questionnaire Star" widely used in the industry. Consent participants could directly fill out the online questionnaire by scanning the QR code or directly clicking the questionnaire star link on the smartphone or tablet device. All relevant information on this study (e.g. research purpose, methods, precautions) was provided with detailed instructions and explanations on the first page the Questionnaire Star. The Electronic Informed Consent page provides two options (yes/no). Only participants who choose “Yes” were directed to the questionnaire completion page.

### Instruments

#### General characteristics

All participants indicated their gender(male, female), age(≤ 18, 19–20, 21–22, 23–24, ≥ 25), grade (freshman, sophomore, junior, senior), place of residence(rural, town, city), student leader or not, be the only child or not, depression or not, anxiety or not, feel lonely or not, the adapting to online class and so on.

#### Athens Insomnia Scale (AIS)

The entire 8-item Athens Insomnia Scale (AIS-8) [21] was developed by Soldatos and colleagues in 2000 to assess the degree of insomnia in subjects based on the International Classification of Diseases Tenth Revision (ICD-10) diagnostic criteria for insomnia. AIS scale is used to assess the insomnia status of participants in the past two weeks. AIS scale contains 8 items, and each item is scored on a 4-point scale(from 0 to3, 0 = no problem, and 3 = very severe problem). The total score are between 0 and 24, the cutoff score of insomnia is 6 [22]. The scale is widely used to screen insomnia. Therefore, the cutoff point used in this study was 7, with a score of > 7 indicating insomnia. The Athens Insomnia Scale showed good internal consistency in this study (Cronbach's α = 0.80).

#### Fear of COVID‐19

The medical college students' fear of COIVD‐19 was measured using the fear of COVID-19 Scale (FCV-19S) [23]. The total FCV-19S sum scores range from 7 to 35, with the higher total score indicating the greater fear of COVID-19. Due to no authoritative severity cutoff for the FCV-19S scale being provided, we used a FCV-19S score of ≥ 21 was used for cutoff points for fear of COVID-19. In the present study, the FCV-19S showed very high reliability, Cronbach’s alpha of 0.83.

#### GAD-2

Anxiety symptoms were assessed using the Generalized Anxiety Disorder Questionnaire-2 (GAD-2). GAD-2 consists of 2 questions, each questionnaire item with a score of 0 to 3. GAD-2 total score ranges from 0 to 6, and a score ≥ 3 is identified as having anxiety symptoms [24]. The GAD-2 showed good internal consistency in this study (Cronbach's α = 0.803).

#### PHQ-2

Depressive symptoms were assessed using the Patient Health Questionnaire-2 (PHQ-2) [25]. PHQ-2 consists of 2 items, each with a score from 0 to 3, and a score ≥ 3 is identified as having depressive symptoms. The PHQ-2 showed good internal consistency in this study (Cronbach's α = 0.813).

### Data analysis

All data analyses were performed using SPSS 20.0 (IBM, New York, USA), and statistical significance was set at *P* < 0.05. Demographic characteristics, insomnia scores, depressive symptoms, anxiety symptoms, and the fear of COVID-19 are presented with mean, standard deviation (SD), numbers and percentages. χ2 tests were used to compare group differences of categorical variables. Binary logistic regression analysis was performed to analyze factors associated with insomnia, and ORs (odds ratios) and 95% CIs (confidence intervals) were calculated.

## Results

### Description of the sample

Table 1 presents the demographic characteristics of the medical college students. Among the 2289 participants, 34.0% (*n* = 779) were male and the rests were female 66.0% (*n* = 1510). The age range of the medical college students was from 17 to 27 years, with a mean of 20.52 ± 1.928. Place of residence was 18.7% (427), 27.7% (633) and 53.7% (1229) for city, town and county, respectively. The number of freshman to fifth-year students was 679(29.7%), 589(25.7%), 454(19.8%), 459(20.1%) and 108(4.7%), respectively. 658(28.7%) were only-child, and 674(29.4%) were student leader.

**Table 1:** Univariate analysis of the participants’ demographic (*N* = 2,289)

Variables	Insomnia (%)			χ^2^	*P*
	**No**	**YES**	**Total**		
	1653(72.2)	636(27.8)	2289(100.0)		
**Gender**				0.063	0.801
**Male**	560(71.9)	219(28.1)	779(34.0)		
**Female**	1093(72.4)	417(27.6)	1510(66.0)		
**Place of residence**				0.062	0.97
**Rural**	890(72.4)	339(27.6)	1229(53.7)		
**Town**	455(71.9)	178(28.1)	633(27.7)		
**City**	308(72.1)	119(27.9)	427(18.7)		
**Loneliness**				184.56	< 0.001
No	1123(82.8)	234(17.2)	1357(59.3)		
**Yes**	530(56.9)	402(43.1)	932(40.7)		
**Adapting to Online Classes**				151.67	< 0.001
**Not adapt**	95(43.6)	123(56.4)	218(9.5)		
**General**	684(67.9)	324(32.1)	1008(44.0)		
**Adapt**	874(82.2)	189(17.8)	1063(46.4)		
**Student leader**				0.191	0.662
**No**	1162(72.0)	453(28.0)	1615(70.6)		
**Yes**	491(72.8)	183(27.2)	674(29.4)		
**The only child**				0.547	0.459
**No**	1185(72.7)	446(27.3)	1631(71.3)		
**Yes**	468(71.1)	190(28.9)	658(28.7)		
**Age**				20.157	< 0.001
** ≤ 18**	217(75.1)	72(24.9)	289(12.6)		
**19–20**	684(72.2)	263(27.8)	947(41.4)		
**21–22**	600(74.3)	207(25.7)	807(35.3)		
**23–24**	106(65.8)	55(34.2)	161(7.0)		
** ≥ 25**	46(54.1)	39(45.9)	85(3.7)		
**Grade**				28.201	< 0.001
**1**	517(76.1)	162(23.9)	679(29.7)		
**2**	428(72.7)	161(27.3)	589(25.7)		
**3**	328(72.2)	126(27.8)	454(19.8)		
**4**	380(67.0)	187(33.0)	567(24.8)		
Depression				280.935	< 0.001
No	1441 (81.2)	334 (18.8)	1589 (69.4)		
Yes	340 (48.6)	360 (51.4)	700 (30.6)		
Anxiety				318.530	< 0.001
No	1358 (82.7)	284 (17.3)	1642 (71.7)		
Yes	295 (45.6)	352 (54.4)	647 (28.3)		

### Factors associated with Insomnia in the univariate analysis

In this study, the prevalence of insomnia among the medical college students was 27.80% (636/2289). There were significant differences between insomnia and loneliness, adapting to online class, depression, anxiety, age, and grade (*P* < 0.01, Table 1). Specifically, feeling lonely, didn't adapting to online class, depression, anxiety, older age and upper grades had more severe insomnia.

### Correlation between the insomnia and fear of COVID‐19 in medical college students

The intercorrelations of our study variables are illustrated in Table 2, the Pearson's correlation coefficient was used to analyze the relationship between insomnia and fear of COVID‐19, and statistically significant positive correlations was found between fear of COVID‐19 and insomnia (*P* < 0.001; *r* = 0.274).

**Table 2:** The correlations between Insomnia and Fear of COVID‐19

Variables	Insomnia	
	**r**	***P* value**
Fear of COVID‐19	0.274^**^	< 0.001

### Factors associated with insomnia

Factors affecting academic burnout of medical college students are shown in Table 3. Insomnia is more severe among students in their third and fourth years. (OR = 1.756, 95% CI 1.188–2.596; OR = 1.679, 95% CI 1.077–2.618). The older the medical college students, the higher the risk of insomnia, and insomnia are the severest among medical students over the age of 25(OR = 2.033, 95% CI 1.012–4.084). fear of COVID‐19、depression、anxiety and loneliness increased the risk of insomnia (OR = 2.043, 95% CI 1.629–2.563, OR = 2.116, 95% CI 1.605–2.789, OR = 2.354, 95% CI 1.778–3.117, OR = 2.558, 95% CI 2.064–3.170). Adapting to online class was a protective factor for insomnia (OR = 0.425, 95% CI 0.304–0.596, OR = 0.217, 95% CI 0.153–0.308).

**Table 3:** Multivariate logistic analysis of factors associated with insomnia

Variable	*B*	*SE*	*Wald*	*P*	OR	95% CI
**Grade (1)**			8.855	0.031		
**2**	0.358	0.161	4.947	0.026	1.431	1.044–1.963
**3**	0.563	0.199	7.971	0.005	1.756	1.188–2.596
**4**	0.518	0.227	5.226	0.022	1.679	1.077–2.618
**Age(≤ 18)**			24.860	0.000		
**19–20**	0.029	0.185	0.025	0.874	1.030	0.716–1.481
**21–22**	-0.450	0.233	3.736	0.053	0.637	0.404–1.006
**23–24**	0.025	0.311	0.006	0.936	1.025	0.558–1.885
** ≥ 25**	0.709	0.356	3.970	0.046	2.033	1.012–4.084
**Fear of COVID‐19**	0.715	0.116	38.203	0.000	2.043	1.629–2.563
depression	0.749	0.141	28.268	0.000	2.116	1.605–2.789
anxiety	0.856	0.143	35.765	0.000	2.354	1.778–3.117
**Loneliness**	0.939	0.109	73.668	0.000	2.558	2.064–3.170
**Adapting to Online Classes**			80.009	0.000		
**General**	-0.855	0.172	24.627	0.000	0.425	0.304–0.596
**Adapt**	-1.527	0.179	72.800	0.000	0.217	0.153–0.308
**Constant**	-1.374	0.221	38.625	0.000	0.253	

## Discussion

In the domestic and international literature, there are few studies have been carried out during the new way of COVID-19 pandemic concerning the insomnia of medical students. To our knowledge, this is the first cross-sectional study conducted to systematically investigate the insomnia status and to explore the related psychological factors among of Chinese medical students who have experienced the new wave of COVID-19 pandemic. Our findings help fills a gap in our understanding of insomnia among medical students during the new wave of the COVID-19 pandemic. The results of this study enrich the research on the incidence of insomnia among medical college students in Anhui in the post-COVID-19 era. Therefore, the findings of this study may contribute to research on insomnia among Chinese medical students and help provide some practical suggestions for reducing the risk of insomnia problems.

Our study shows that the COVID-19 pandemic had a significant impact on the insomnia of Chinese medical college students. In our study, the prevalence of insomnia among medical students during this the new wave of COVID-19 pandemic was found to be 27.8%. The prevalence of insomnia symptoms of China medical college students was significantly higher than in the non-epidemic period (18.5%) [5] and previous study in Bangladesh [26], lower than the COVID-19 lockdown period [27, 28]. State Insomnia was reported by 27.8% of the participants, which may reflect the prevalence of insomnia among Chinese medical students after the COVID-19 pandemic. Feelings of loneliness, vulnerability, and worry caused by the COVID-19 pandemics and social isolation can trigger an increase in anxiety and depressive symptoms in medical students. Anxiety and fear can increase cortisol levels, decreased melatonin synthesis, and decreased sleep quality, with changes in the biological rhythms [29, 30]. At the same time, it has been suggested that dysregulation of the hypothalamic–pituitary–adrenal axis may be related to the relationship between sleep deprivation and feelings of loneliness and fear [31, 32].

The results of this study have very important clinical and public health implications. The possible cause of insomnia may be related to the rapid spread of COVID-19 and the perceived stress of study and life. The differences in the prevalence of insomnia among medical students in different studies could be partly explained by the different study periods and the use of survey instruments. In addition, different school types, grades, majors, research objects and social environments could also affect the occurrence of insomnia among college students. Combining all the above analysis results, this study has strong reasons to suggest that more attention is needed to medical students' insomnia in the context of the COVID-19 pandemic. Therefore, active coping strategies should be developed to prevent insomnia in medical students.

As expected, older age was positively associated with insomnia, reaffirming that age is an important risk factor for insomnia [33]. And, the results of this study showed that medical colleges students in higher grades had a higher prevalence of insomnia compared with those in the lower grades, which confirms previous findings. Because final-year college students in China are transitioning from college to society, they have more mental health problems than their peers. University in China, especially in the final year, is an important turning point, and various pressures such as graduation and employment are involved [34]. Another explanation may be that severely affected by the COVID-19 outbreak, colleges and universities have cancelled classroom teaching, students had to study online in dormitories, and graduates are unable to find jobs in time, which all contribute to the occurrence of insomnia. Additionally, the results remind the medical school staff and the administrative people to raise awareness among seniors medical college students of good sleep hygiene.

In the early phase of the COVID-19 pandemic, UNESCO stated that 1.5 billion students in 188 countries around the world will face a huge fear of being out of school due to the home isolation policies resulting from long-term lockdowns [35]. Online learning was considered to be the main way students learn during the pandemic. With the increasing impact of the COVID-19 pandemic, online learning has become the norm. Study found that online (distance) learning had a direct and long-term impact on college students' physiology, psychology, and lives [36, 37]. Study found that the effectiveness of online learning was varied, also the effectiveness could be influenced by students' own characteristics, such as gender, attitude [38], satisfaction [39], learning style [40] and participation [41]. Prolonged exposure to digital devices can increase other stressors associated with isolation times and lockdown, ultimately leading to exhaustion and burnout [42]. A study showed that online learning increased the psychological stress of Chinese college students [43]. The proliferation of online learning led students to spend more time facing screens and smart phones. Long duration of online learning can lead to physical problems for college students, such as eye strain, cervical stiff, dizziness, headache, insomnia, etc. Study found that increased computer usage among teenagers was associated with increased levels of anxiety [44], and increased online activity was associated with moderate-to-severe depression [45]. Medical students with greater adaptability showed more active learning enthusiasm and greater learning engagement in online learning [46] The high satisfaction with online learning can stimulate students' sense of achievement and adaptability in the online learning process, improve learning efficiency, and effectively alleviate their psychological pressure and reduce insomnia. As a result, teachers can take flexible measures to stimulate students' interest and enthusiasm for online learning, create an atmosphere of positive communication, and increase students' participation in online learning.

Another key finding is that during the new wave of COVID-19 pandemic, Fear of COVID‐19 among medical college students was positively correlated with insomnia. Overall, our findings were consistent with our research hypothesis. The highly contagious nature of COVID-19 and the fact that it has serious consequences may lead to fear COVID‐19 among medical students [47]. Previous research indicated that the COVID-19 pandemic and dramatic changes in learning styles pose significant threats to medical students' physical and mental health, such as fear of the COVID-19 pandemic. The further development of COVID-19 fear may lead to the occurrence of stress, depression, anxiety psychological disorders and endocrine disorders, growing fears of COVID-19 can trigger a range of serious physical dysfunctions, such as insomnia [17]. Research had shown that fears of COVID-19, including fear of the future and fear of contracting the COVID-19, as well as concerns about their own health, which is linked to poor mental health problems [48]. In accordance with the Chinese government epidemic prevention policy on COVID-19, strict regulations have been issued for college students in China to prevent the spread of COVID-19 in the university community. The situation has created panic among medical students, especially those living in cities with the COVID-19 pandemic. This finding suggests that reducing COVID-19 fear among medical students may be a good strategy for reducing stress and improving their insomnia among medical students during the new COVID-19 pandemic. Therefore, reducing the fear of COVID-19 could help medical students to perceive less stress. It can be considered an unavoidable result that fear of COVID‐19 leads to insomnia problems among medical students. Therefore, college administrators should not ignore the fears of COVID-19 among medical students during the COVID‐19 pandemic. The exact mechanism between fear of COVID‐19 and insomnia deserves further study.

We also found that anxiety and depression were also significant positive associated with insomnia among Chinese medical college students, the results of this study was similar to those of previous studies [49]. After binary logistic regression analysis, we found that anxiety and depression may be a potential risk factor for insomnia in medical students. Hosen et al. [50] reported the symptoms of depression (43.3%) and anxiety (32.6%) among students in Bangladesh during the COVID-19 outbreak. Previous study [51] has shown that the HPA axis is thought to be the physiological link between mental health and insomnia problems. Therefore, medical college students with anxiety and depression often experience insomnia problems due to circadian rhythms and the HPA axis. A study by Zhao Y et al. [52]a structural equation modeling found that uncertainty about the COVID-19 pandemic is closely related to anxiety, depression sensitivity, and in turn affects insomnia through depression and anxiety. It cannot be ignored that due to the cross-sectional design of this study, this study did not demonstrate the causal relationship between insomnia and depression. The possible reasons for the correlation between insomnia and depression and anxiety may include the psychological impact of the COVID-19 pandemic on medical students, and factors of COVID-19 pandemic stress.

We also found that loneliness was independently associated with insomnia in medical college students, which was consistent with findings prior to the COVID-19 pandemic. Loneliness was defined as a negative emotional state, a negative feeling of being isolated [53]. Prior research demonstrating that the relationship between loneliness and insomnia is bidirectional [36], therefore loneliness provides an important foundation for clinical insomnia among medical college students during the COVID-19 pandemic. In the context of the COVID-19 pandemic, this study indicates that greater loneliness is positively associated with greater insomnia among medical college students. With the current rollout of strict social distancing measures to control the COVID-19 pandemic, loneliness had become a prominent public health problem, especially among medical college students. The management of strict COVID-19 quarantine measures never experienced before has led to the gradual distancing of medical college students, resulting in social isolation. Lack of interpersonal communication can also cause or exacerbate loneliness.

## Limitations

This paper investigates college medical students' insomnia and the influencing factors in China under the COVID-19 pandemic. The limitations of this study are as follows. The first is that all information was based on self-reported symptoms among medical college students. We did not obtain data from other sources. Secondly, only one institution was surveyed in this study, which might not to represent all medical students in China. Thirdly, this was a cross-sectional study, so it was not designed to investigate changes in insomnia over time. Finally, it was not possible to assess the causal relationship between COVID-19 fear, mental health, and insomnia because all variables were assessed simultaneously.

## Conclusions

The current study demonstrated that the Chinese medical college students experienced a certain degree of insomnia during the COVID‐19 pandemic. Grade, age, fear of COVID‐19, depression, anxiety, loneliness, and adapting to online class could affect insomnia in Chinese medical students. The results of the current study remind us is that even after the COVID-19 pandemic, it is necessary to conduct more cross-sectional investigations into the causes and consequences of insomnia among medical students. Moreover, school administrators should take effective intervention measures to prevent the insomnia in medical students. With this work, we provide clues to early identification of sleep problems with medical students and help to act preventively.

## Data Availability

The datasets in the current study of this study are available from the corresponding author on reasonable request.
